# Three plant growth-promoting rhizobacteria from the Qinghai–Tibet Plateau enhanced plant growth and alleviated low-temperature and drought stress

**DOI:** 10.3389/fpls.2026.1907342

**Published:** 2026-09-03

**Authors:** Qiubei Chen, Yuxuan Wang, Huali Luo, Nima Genqing, Ke Tao

**Affiliations:** 1 Key Laboratory of Bio-Resource and Eco-Environment of Ministry of Education, College of Life Sciences, Sichuan University, Chengdu, Sichuan, China; 2 Forestry and Grassland Bureau of Shiqu County, Ganzi, China

**Keywords:** alpine meadow, cold tolerance, drought tolerance, *Lamiophlomis rotata*, plant growth-promoting rhizobacteria (PGPR), Qinghai-Tibet Plateau, whole-genome sequencing

## Abstract

**Introduction:**

Degradation of alpine meadows on the Qinghai-Tibet Plateau has severely restricted regional livestock development and ecological security. Exploring rhizosphere microbial resources with growth promoting and stress alleviating functions is an effective strategy for promoting grassland restoration.

**Methods:**

In this study, three PGPR strains were isolated from the rhizosphere of *Lamiophlomis rotata*, a dominant species in degraded alpine meadows, and identified as *Pseudomonas* sp. YD4-3, *Pseudomonas* sp. YD4-4, and *Lelliottia* sp. ND3-5. Their effects on *Elymus dahuricus* were evaluated through germination pouch assay, low-temperature pot experiment and drought stress experiment. Whole-genome sequencing was performed on strain YD4-4, which showed the strongest effects in the experiments.

**Results:**

All three strains promoted biomass accumulation and significantly improved low-temperature and drought tolerance. Strain YD4-4 exhibited the greatest effects. Whole-genome sequencing predicted that YD4-4 harbors multiple functional genes related to cold adaptation, IAA biosynthesis, nutrient transformation, and oxidative stress resistance. Genomic analyses further suggested that YD4-4 may represent a novel species within the genus *Pseudomonas*.

**Discussion:**

This study provides microbial resources for alpine meadow restoration and the development of new biofertilizers. The genomic data of YD4-4 offer a basis for understanding how this strain promotes plant growth and alleviates stress, which may inform future strain improvement and field applications.

## 1 Introduction

Alpine meadows represent a dominant vegetation type on the Qinghai-Tibet Plateau (Duan et al., 2021). They support local livelihoods and provide key ecological functions including biodiversity conservation and nutrient cycling (Feng and Squires, 2020; Li et al., 2021; Bai-ping et al., 2002), thereby serving as one of the most important grassland ecosystems globally (Zhou et al., 2023). However, under the plateau’s unique topography and climate, grassland growth is constrained by uneven precipitation, drought and low-temperatures (Swemmer et al., 2007; Li et al., 2024; Wingler and Hennessy, 2016). Data shows that arid and semi-arid areas account for 67% of the total area of the Qinghai-Tibet Plateau (Wang et al., 2013). In recent years, soil degradation from overgrazing and other anthropogenic activities has further reduced forage quality, limiting sustainable livestock development (Yang and Sun, 2021; Yan et al., 2024; Song, 2025).

Fertilization is commonly used to restore degraded alpine meadows (Zong et al., 2021). The application of fertilizers, especially nitrogen fertilization, can significantly improve plant health in degraded grasslands and shorten restoration time (Sheng et al., 2022). Unlike chemical fertilizers that risk environmental pollution, plant growth-promoting rhizobacteria (PGPR) naturally inhabiting alpine meadow rhizospheres can be developed into ecofriendly biofertilizers (Shahzad et al., 2025). PGPR promote host growth and stress tolerance through multiple synergistic mechanisms. For nutrient improvement, PGPR convert soil nitrogen, phosphorus, and potassium into plant-accessible forms via biological nitrogen fixation, phosphate solubilization, and potassium mobilization, enhancing nutrient use efficiency (Zhiyong et al., 2024; Jia et al., 2026; Sattar et al., 2019). For phytohormone regulation, PGPR synthesize auxins like IAA, promoting root development and nutrient uptake by modulating endogenous phytohormone balance (Olagunju et al., 2026; Ahmad et al., 2022; Buqori et al., 2025). PGPR also alleviate abiotic stresses including low temperature and drought by activating plant antioxidant systems, maintaining photosynthetic organ integrity, and inducing osmotic regulators, enhancing host adaptability (Su et al., 2015; Sharma et al., 2025).

However, PGPR have mostly been developed for mild conditions (Yang et al., 2022; Mellidou and Karamanoli, 2022; Basu et al., 2021). Their colonization and efficacy are often unstable under extreme plateau conditions like cold and drought (Kumari et al., 2024; El-Saadony et al., 2024), limiting their large-scale application in alpine grassland restoration. Therefore, screening efficient PGPR strains adapted to extreme environments from native plateau plant rhizospheres is important for developing specialized biofertilizers suitable for alpine meadows (El-Saadony et al., 2024; Chai et al., 2023). Plants thriving in extreme environments often form symbiotic associations with stress-tolerant microorganisms, making their rhizosphere a promising source of PGPR adapted to harsh conditions (Rajasreelatha and Thippeswamy, 2022; Staiano et al., 2025).

*Lamiophlomis rotata*, as a dominant species in degraded grasslands of the Qinghai-Tibet Plateau (Niu et al., 2020), has evolved multiple stress-resistance mechanisms through long-term adaptation to cold, drought, and poor soils (Xu et al., 2025; Wang et al., 2025). Morphologically and physiologically, *L. rotata* improves the rhizosphere microenvironment via root-secreted organic compounds or regulate leaf structural traits to enhance photosynthesis (Niu et al., 2020; Zhang et al., 2024). At cellular and molecular levels, *L. rotata* adapts to high-altitude environments by upregulating antioxidant enzyme activities and accumulating osmotic regulators (e.g., proline and soluble sugars) (Jie et al., 2020). Furthermore, proteomic and transcriptomic analyses have revealed its molecular adaptation strategies, in which H_2_S acts as a key signaling molecule that regulates high-altitude adaptation by activating antioxidant defenses and mitigating reactive oxygen species (ROS) and reactive nitrogen species (RNS) damage (Ma et al., 2015). These stress-resistance mechanisms suggest that the *L. rotata* rhizosphere may harbor PGPR with exceptional environmental adaptability. However, current studies on the *L. rotata* rhizosphere microbiota have largely been limited to high-throughput sequencing-based community structure analyses (Wu et al., 2025; Qiao et al., 2023). Systematic investigations on isolation, screening, and mechanisms of functional bacterial strains remain insufficient.

With the rapid development of high-throughput sequencing technologies, whole-genome sequencing has become a key tool for dissecting PGPR functional mechanisms, overcoming limitations of traditional phenotypic methods and revealing their genetic basis at the molecular level (Paterson et al., 2017; Gupta et al., 2014). Whole-genome approaches strongly predict functional genes for nitrogen fixation, phosphate solubilization, phytohormone synthesis, and siderophore production (Blake et al., 2025; González-Cruz et al., 2025), and biosynthetic gene clusters for secondary metabolites involved in growth promotion and disease suppression (Dhanalakshmi and Rajendhran, 2024; Nielsen et al., 2021), showing that it’s effective and reliable for characterizing PGPR-mediated growth promotion and application potential. However, PGPR from extreme environments (e.g., arid and alpine regions) remain underexplored (Jin et al., 2025; Guo et al., 2025). Therefore, systematic whole-genome sequencing and integrative analysis of PGPR from alpine habitats are urgently needed to uncover microbial adaptive mechanisms and discover novel PGPR resources.

Using rhizosphere soil of *L. rotata* from the Qinghai-Tibet Plateau, this study isolated, screened, and taxonomically identified three PGPR strains and evaluated their functions including phosphate solubilization, nitrogen fixation, potassium mobilization, IAA production, and cold tolerance. Furthermore, using Elymus dahuricus, an important forage grass in alpine meadows, as the host, the growth-promoting and stress tolerance-inducing effects of the three strains were validated through germination pouch assays, low-temperature pot experiment, and drought stress experiment. Meanwhile, whole-genome sequencing and functional annotation were performed on the best-performing strain YD4–4 to provide insights into the genetic basis of its growth-promoting mechanisms. This study provides high-quality microbial strain resources for remediating degraded alpine grasslands and establishes a theoretical foundation for developing novel, ecofriendly microbial inoculants for the Qinghai-Tibet Plateau.

## 2 Materials and methods

### 2.1 Sample collection

Soil samples were collected from Shiqu County, Ganzi Tibetan Autonomous Prefecture, Sichuan, China (33°04′11″N, 97°58′33″E; 4166.11 m a.s.l.). Sampling was conducted in two distinct types of degraded grassland habitats: a toxic weed area and a rodent-affected barren area. Four *L. rotata* plants were selected from each habitat, with ≥10 m between sampling points. After removing surface debris, the whole plant including the rhizosphere soil was carefully excavated using a shovel sterilized with 75% ethanol. All samples were placed into sterile zip-lock bags and stored at 4 °C.

### 2.2 PGPR isolation and purification

Ten grams of rhizosphere soil from each sample was separately suspended in 100 mL of sterile water in a flask and placed on a rotary shaker at 120 rpm and 28°C for 1 h and the suspension was serially diluted to 10^−4^, 10^−5^, and 10^−6^. Each dilution was spread onto plates of Pikovskaya (PKO) agar (Sang et al., 2022), followed by incubation at 28 °C for 7 days. Colonies exhibiting transparent zones were selected and repeatedly streaked on PKO agar plates until morphologically stable single colonies were obtained. Isolates with larger transparent zones were chosen for subsequent experiments.

### 2.3 Assessment of plant growth-promoting traits of the PGPR isolates

Each strain was inoculated at 1% (v/v) into PKO medium and King’s medium (Feng et al., 2024) and incubated at 28 °C with shaking at 140 rpm for 7 days (PKO) or 3 days (King’s). The soluble phosphorus was determined by the molybdenum blue method following the tissue inorganic phosphorus assay kit instructions (Solarbio, Beijing, China). IAA secretion was measured by the Salkowski method (Gang et al., 2019). Additionally, the strains were spot-inoculated onto nitrogen-free agar and Aleksandrov agar (Bagyalakshmi et al., 2017) and incubated at 28 °C for 7 days to assess nitrogen fixation and potassium mobilization. For cold tolerance, strains were cultured on LB agar at 4 °C for 48 h, and growth was evaluated by colony formation.

### 2.4 Characterization and identification of the PGPR isolates

Cellular morphology of the three pure isolates was examined by transmission electron microscopy (TEM). Electron micrographs were obtained commercially from Li Lai Biotechnology Co., Ltd., Chengdu, China. Genomic DNA was extracted using a bacterial genomic DNA extraction kit (Tiangen, Beijing, China). The 16S rDNA gene was amplified with the universal primers 27F (5’-AGAGTTTGATCCTGGCTCAG-3’) and 1492R (5’-GGTTACCTTGTTACGACTT-3’). Each PCR reaction mixture (25.0 μL) contained 12.5 μL of Q5 High-Fidelity 2X Master Mix, 1.25 μL of each primer (10 μM), 2.0 μL of DNA template, and 8.0 μL of nuclease-free water. The PCR conditions were 30 cycles of initial denaturation at 95 °C for 30s,denaturation at 94 °C for 10 s, annealing at 58 °C for 30 s, and extension at 72 °C for 45 s, followed by a final extension at 72 °C for 5 min. After verification of the PCR products by electrophoresis on a 1.5% agarose gel, the amplicons were sent to Personal Biotechnology Co., Ltd., Shanghai, China for sequencing. A phylogenetic tree was generated via the neighbor-joining algorithm implemented in MEGA 12 software. Sequences were aligned and compared against NCBI database sequences for phylogenetic placement (Zhang et al., 2025).

### 2.5 Seed germination pouch assay

Three activated strains were inoculated into prepared LB liquid medium at a 1% (v/v) inoculum and cultured at 28 °C with shaking at 180 rpm for 12 h and the bacterial suspensions were diluted with sterile water to OD_600_ = 0.8. *E. dahuricus* seeds (Jiangsu Gangrenboqi Seed Co., Ltd., Jiangsu, China) were surface sterilized with 2% NaClO for 10 min followed by 70% ethanol for 3 min, and rinsed continuously with sterile water for more than 10 times until there is no residual taste of NaClO. Seeds were soaked in the corresponding bacterial broth (LB medium as control) for 5 min. They were then sown on Petri dishes containing moistened, sterile filter paper for germination at 28 °C. Uniform seedlings were transferred to germination pouches moistened with sterile water. Plants were grown in a growth chamber for 14 days at 28/20 °C day/night, 16/8 h light/dark photoperiod, and 20,000 lx light intensity, with sterile water replenished every other day. After cultivation, measure the length, fresh weight, and dry weight of shoots and roots (He et al., 2018). Each treatment included 12 replicates.

### 2.6 Low-temperature pot experiment and drought stress experiment

Each pot was filled with 1000 g of an autoclaved mixture of fine sand and vermiculite (1:1, v/v) and sown with 2.5 g of surface-sterilized seeds. Growth conditions were 10 °C, 14/10 h light/dark photoperiod, and 10,000 lx light intensity, simulating June climate on the Qinghai-Tibet Plateau (He M. et al., 2021). Watering was performed every 3 days. After emergence, treatment groups received 10 mL fermented bacterial broth (OD_600_ = 0.8), and controls received 10 mL LB liquid medium. Each treatment included 6 pots and was randomly placed in the artificial climate chamber. Plants were cultivated for 40 days, then sampled for shoot biomass, chlorophyll (He A. et al., 2021), root activity (BC5275, Solarbio, Beijing, China), and total nitrogen (Novamsky et al., 1974) and phosphorus (BC2855, Solarbio, Beijing, China).

The drought stress experiment was conducted in the same manner as above. Each treatment included 6 pots and was randomly placed in the artificial climate chamber. After 40 days of normal cultivation, watering was stopped ofor 15 days of natural drought. Samples were analyzed for chlorophyll (He A. et al., 2021), activities of superoxide dismutase (SOD) (BC0175, Solarbio, Beijing, China), peroxidase (POD) (BC0095), catalase (CAT) (BC0205), proline (Pro) (BC0295), and malondialdehyde (MDA) (BC0025).

### 2.7 Complete genome sequencing and annotation

Strain YD4–4 was inoculated at 1% into 500 mL of LB liquid medium and cultured at 28 °C with shaking at 140 rpm for 16 h. The fermentation broth was centrifuged at 8000 ×g for 10 min at 4 °C to harvest the cells. After discarding the supernatant, cells were resuspended and washed with 1× PBS buffer, then centrifuged 1–2 times until the supernatant was clear. The final cell pellet was stored on dry ice and sent to Shanghai Personal Biotechnology Co., Ltd. for whole-genome sequencing.

### 2.8 Statistical analysis

Growth and physiological data are expressed as mean ± standard error. Statistical analysis was performed using SPSS 27 (SPSS Inc., Chicago, IL, USA). Differences among means were assessed by one-way analysis of variance (ANOVA) followed by Duncan’s multiple comparison test, with statistical significance set at P < 0.05. The normality (*P* < 0.05) of all data and the homogeneity of variance (*P* < 0.05) were confirmed through the Shapiro–Wilk test and Levene’s test, respectively, using SPSS 27. All experimental procedures were conducted with a minimum of 3 independent replicates.

## 3 Results

### 3.1 Isolation of PGPR from *L. rotata* roots

Screening for growth promoting traits yielded three bacterial strains from the *L. rotata* rhizosphere (**Table 1**). YD4–4 exhibited superior plant growth-promoting traits compared to YD4–3 and ND3-5. It showed the highest inorganic phosphate solubilization (12.15 ± 0.30 mmol/L), IAA production (22.19 ± 1.46 mg/L), and potassium mobilization (D/d ratio of 3.59 ± 0.12), while also testing positive for nitrogen fixation and cold tolerance. YD4–4 thus possesses the most comprehensive growth promoting potential among the three isolates.

**Table 1:** Plant growth promoting characteristics of three strains.

Strains	Phosphate solubilization (mmol/L)	IAA production (mg/L)	Potassium mobilization (D/d)	Nitrogen fixation	Cold tolerance
YD4-3	8.71 ± 0.08	2.37 ± 0.26	3.33 ± 0.16	–	+
YD4-4	12.15 ± 0.30	22.19 ± 1.46	3.59 ± 0.12	+	+
ND3-5	1.01 ± 0.05	10.66 ± 1.36	–	+	+

D denotes the halo diameter, and d denotes the colony diameter. Data represent the mean ± standard deviation.

### 3.2 Characterization and identification of three PGPR isolates

**Figure 1:**
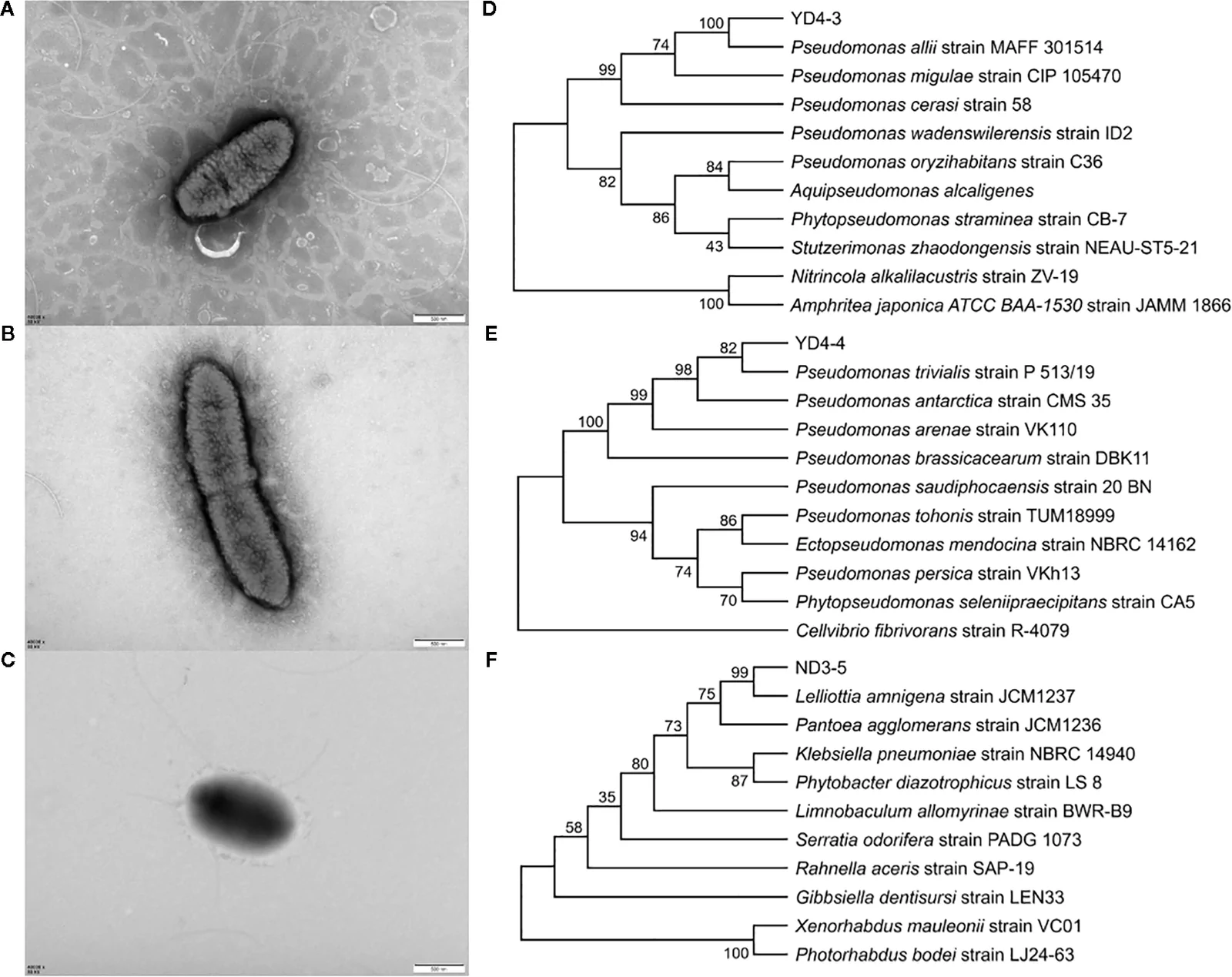
Morphological characteristics and phylogenetic analysis of three strains. **(A–C)** TEM images of strain YD4-3, YD4–4 and ND3-5. **(D–F)** Phylogenetic tree based on 16S rDNA gene sequences. The numbers on the branches represent the support rate, with the maximum value set at 100.

TEM micrographs showed that YD4-3, YD4-4, and ND3–5 were rod shaped and flagellated (**Figures 1A–C**). YD4–3 was a bacillus with bluntly rounded ends, approximately 1.7 μm × 0.8 μm. YD4–4 was a straight, elongated rod, approximately 2.2 μm × 0.8 μm. ND3-5 was nearly ovoid, approximately 1.1 μm × 0.7 μm. Based on 16S rDNA sequencing, YD4–3 and YD4–4 belong to *Pseudomonas*, and ND3–5 belongs to *Lelliottia* (**Figures 1D–F**). Sequencing data are deposited in NCBI under GenBank accession numbers PZ225623, PZ225624, and PZ225625.

### 3.3 The effects of three PGPR strains on plant growth of *E. dahuricus*

#### 3.3.1 Permission to reuse and copyright

In the seed germination pouch experiment, *E. dahuricus* seedlings inoculated with YD4-3, YD4-4, and ND3–5 exhibited significantly higher aboveground and belowground biomass compared to the control group (**Table 2**). Both shoot and root length were significantly greater in the inoculated groups (*P* < 0.05), with the most pronounced increases observed in ND3-5-inoculated seedlings, reaching 5.40 ± 1.08 cm and 16.89 ± 1.97 cm. Inoculated seedlings showed increased trends in both fresh and dry weight of shoots, while the fresh and dry weights of roots were significantly enhanced (*P* < 0.05). YD4-3-inoculated seedlings showed the highest fresh weight increases: shoot 9.31 ± 2.02 mg and root 8.23 ± 4.92 mg. For dry weight, ND3–5 gave the largest shoot increase (2.32 ± 0.80 mg), and YD4–3 gave the largest root dry weight (2.05 ± 0.23 mg). Thus, all three strains are potential PGPR candidates for promoting *E. dahuricus* growth, with strain specific effects.

**Table 2:** Results of seed germination pouch experiment.

Parameter		LB	YD4-3	YD4-4	ND3-5
Shoot	Length (cm)	3.61 ± 0.85a	5.25 ± 0.72b	4.78 ± 0.78b	5.40 ± 1.08b
	Fresh Weight (mg)	6.38 ± 2.38a	9.31 ± 2.02b	8.15 ± 2.01ab	9.13 ± 3.05b
	Dry Weight (mg)	1.53 ± 0.53a	2.22 ± 0.43b	1.92 ± 0.57ab	2.32 ± 0.80b
Root	Length (cm)	11.09 ± 2.95c	16.08 ± 1.21ab	14.57 ± 2.35b	16.89 ± 1.97a
	Fresh Weight (mg)	1.54 ± 0.62a	8.23 ± 4.92b	5.68 ± 4.37b	5.37 ± 2.98b
	Dry Weight (mg)	0.93 ± 0.39c	2.05 ± 0.23a	1.75 ± 0.57b	1.86 ± 0.62a

LB represents the control group, and the rest are the treatment groups inoculated with the corresponding strains. Data represent the mean ± standard deviation. Different letters indicate significant differences (*P* < 0.05, using ANOVA followed by Duncan test).

#### 3.3.2 Low-temperature pot experiment

**Figure 2:**
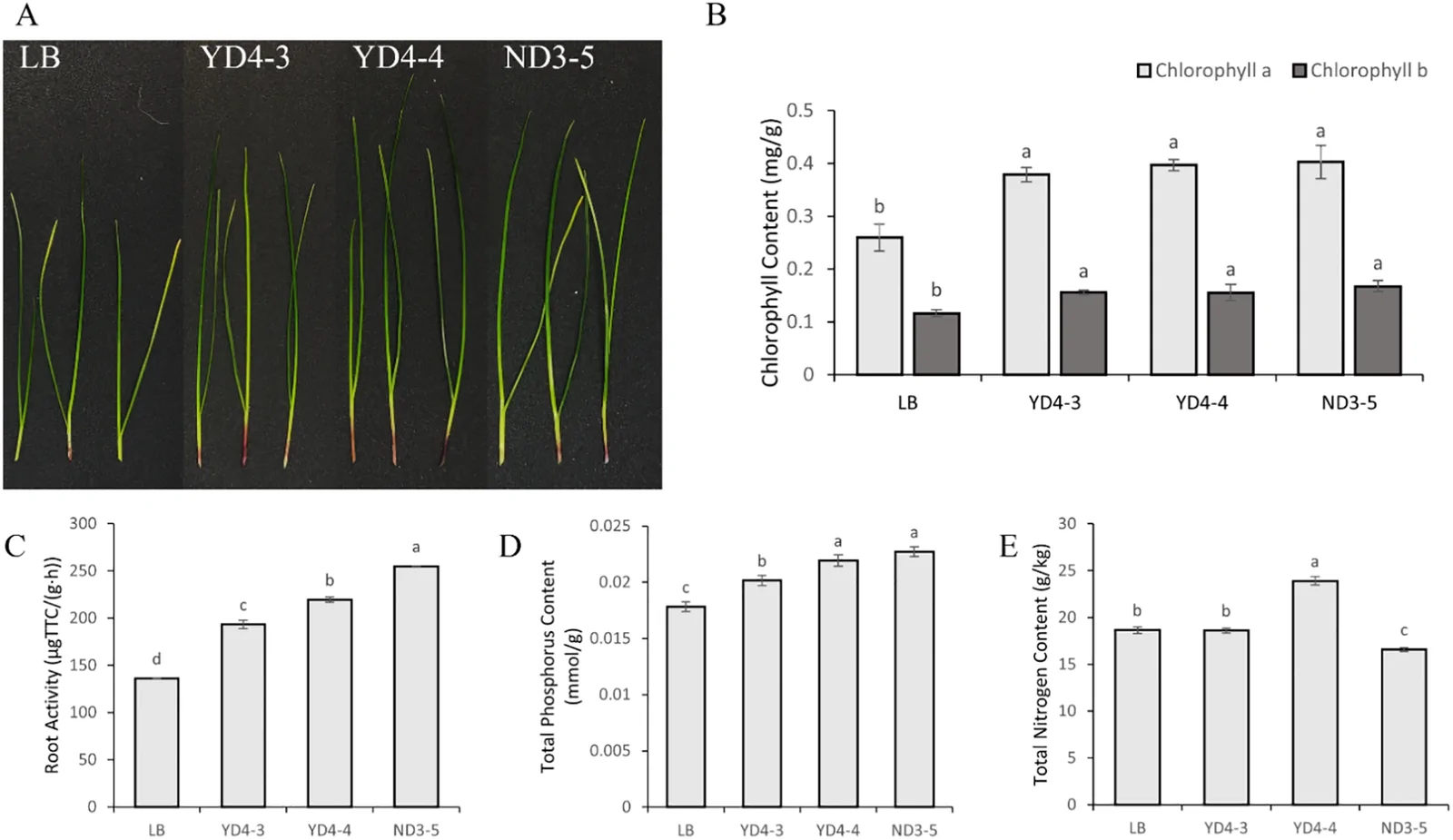
Results of low-temperature pot experiment. **(A)** *E. dahuricus* inoculated with LB, YD4-3, YD4-4, or ND3-5. **(B)** Chlorophyll content. **(C)** Root activity. **(D)** Total phosphorus content. **(E)** Total nitrogen content. LB represents the control group, and the rest are the treatment groups inoculated with the corresponding strains. Data represent the mean ± standard deviation. Different letters indicate significant differences (*P* < 0.05, using ANOVA followed by Duncan test).

In the pot experiment, *E. dahuricus* inoculated with YD4-3, YD4-4, or ND3–5 showed significantly greater aboveground biomass (plant height, fresh weight, dry weight) than controls (**Figure 2A**). YD4-4 showed the strongest effects: height 9.00 ± 0.61 cm, fresh weight 29.00 ± 4.80 mg, dry weight 6.30 ± 1.30 mg (**Table 3**).

**Table 3:** Results of low-temperature pot experiment.

Parameter	LB	YD4-3	YD4-4	ND3-5
Length (cm)	6.41 ± 0.85c	7.91 ± 0.55b	9.00 ± 0.61a	8.93 ± 0.63a
Fresh Weight (mg)	15.00 ± 4.00c	23.00 ± 3.50b	29.00 ± 4.80a	29.00 ± 5.60a
Dry Weight (mg)	3.50 ± 1.00c	5.5 ± 1.10b	6.30 ± 1.30a	6.20 ± 1.60a

LB represents the control group, and the rest are the treatment groups inoculated with the corresponding strains. Data represent the mean ± standard deviation. Different letters indicate significant differences (*P* < 0.05, using ANOVA followed by Duncan test).

Inoculation with YD4-3, YD4-4, or ND3–5 also significantly enhanced: chlorophyll a, chlorophyll b, root activity and total phosphorus content (**Figures 2B–D**). However, only YD4–4 significantly elevated total nitrogen; YD4–3 and ND3–5 had no significant effect (**Figure 2E**). Based on its superior performance in enhancing height, biomass, chlorophyll, and nutrients under pot conditions, YD4–4 was selected for further mechanistic studies.

#### 3.3.3 Drought stress experiment

**Figure 3:**
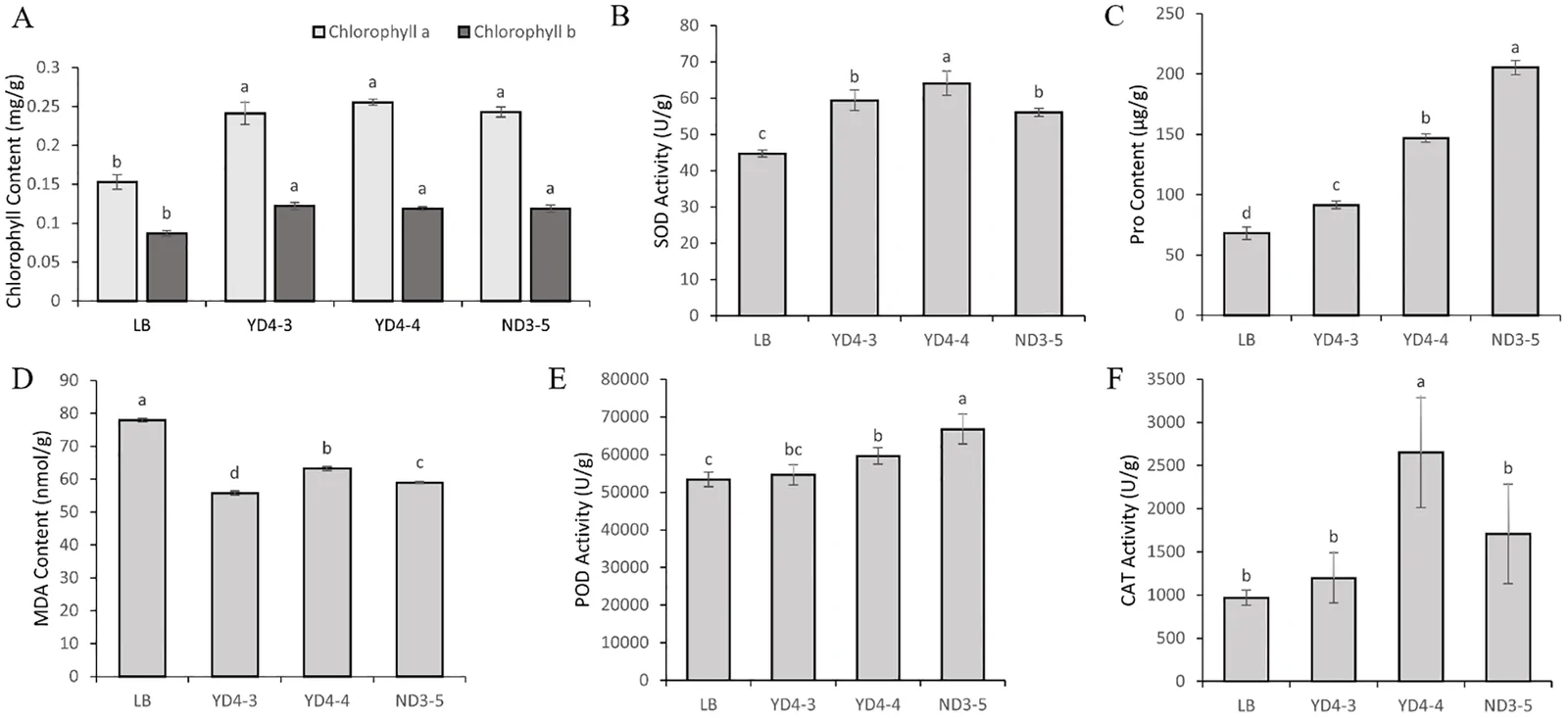
Results of drought stress experiment. **(A)** Chlorophyll content. **(B)** SOD activity. **(C)** Pro content. **(D)** MDA content. **(E)** POD activity. **(F)** CAT activity. LB represents the control group, and the rest are the treatment groups inoculated with the corresponding strains. Different letters indicate significant differences (*P* < 0.05, using ANOVA followed by Duncan test).

Under drought stress, inoculation with strains YD4-3, YD4-4, and ND3–5 significantly influenced the physiological responses of *E. dahuricus*. Inoculated plants showed significant increases in chlorophyll a and chlorophyll b (**Figure 3A**). Regarding the antioxidant system, all inoculations significantly enhanced SOD activity and Pro content, and reduced MDA content (**Figures 3B–D**). For POD activity, only YD4–4 and ND3–5 significantly increased it (**Figure 3E**). CAT activity was significantly enhanced only by YD4-4 (**Figure 3F**); increases with YD4–3 and ND3–5 were not significant. Thus, YD4–4 showed the strongest protective effects against drought stress, supporting its selection for mechanistic studies.

### 3.4 The effects of three PGPR strains on plant growth of *E. dahuricus*

**Figure 4:**
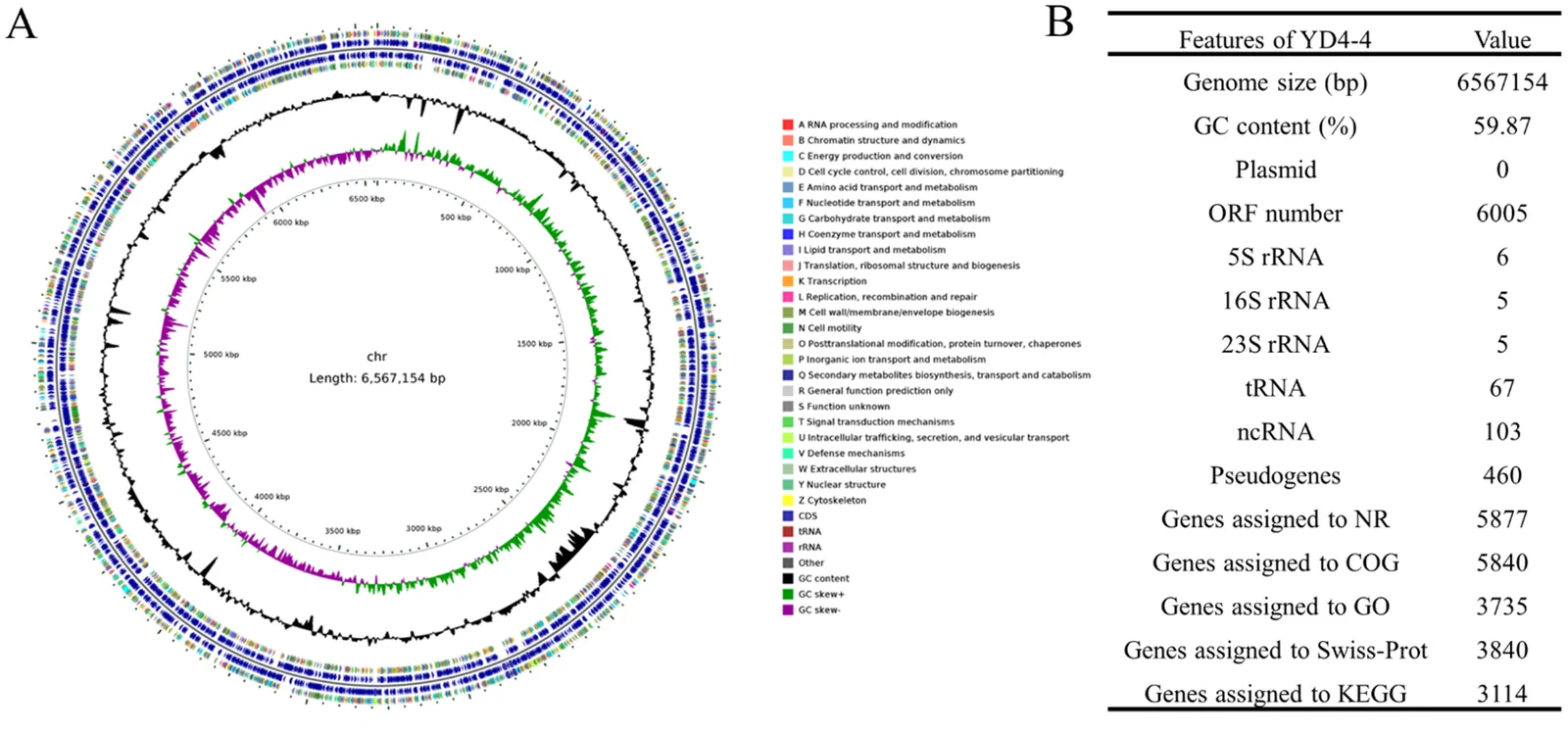
Genomic characteristics of YD4-4. **(A)** Circular genome map of YD4-4. From the inside to the outside, the first circle represents the scale; the second circle represents GC Skew; the third circle represents GC content; the fourth and seventh circles represent the COG to which each CDS belongs; the fifth and sixth circles represent the positions of CDS, tRNA and rRNA on the genome. **(B)** Genome annotation summary of YD4-4.

The YD4–4 genome comprises a single circular chromosome of 6,567,154 bp (**Figure 4A**), with 59.87% GC content (**Figure 4B**). A total of 6,005 protein-coding genes were predicted. The chromosome harbors 67 tRNA genes, 16 rRNA genes (6 5S, 5 16S, 5 23S), and 103 ncRNA genes. For functional characterization, 5,877 genes were assigned to NR, 5,840 to COG, 3,735 to GO, 3,840 to Swiss-Prot and 3,114 genes to KEGG. The ANI analysis conducted using fastANI showed that *Pseudomonas tritici* had the highest ANI value (95.267%) with YD4-4. Further calculations revealed an ANIb value of 94.308% and a dDDH value of 61.2% between YD4–4 and *Pseudomonas tritici.*

Based on whole-genome sequencing, YD4-4 harbors multiple functional gene classes associated with plant growth promotion (**Supplementary Table 1**). These genes are involved in stress response, hormone synthesis/metabolism, and nutrient transformation/uptake, providing a theoretical foundation for its applications.

The identification of low-temperature adaptation-related genes imply the strain’s potential mechanism for coping with cold environments. The genome contains several cold shock protein-coding genes (e.g., *cspA*, *capB*, *cspD*) (Keto-Timonen et al., 2016; Panicker et al., 2010), which help maintain mRNA structural stability under low-temperature conditions (Jalava et al., 2006), ensuring the normal synthesis of essential proteins (Pärn et al., 2015). Additionally, genes related to unsaturated fatty acid synthesis, such as the *fab* gene family (Rosenfeld et al., 1973; Feng and Cronan, 2011) and *desA* (Zhu et al., 2006), regulate the lipid composition of the cell membrane (Hagve, 1988), enhancing its fluidity at low-temperatures (Harayama and Antonny, 2023). Furthermore, osmotic regulation system genes *beta/B* (Yang Y. et al., 2025) (control betaine synthesis) and their corresponding transporter gene cluster *opu* (Hoffmann et al., 2013; Kappes et al., 1996), along with the global regulator *rpoS* (Hengge-Aronis et al., 1993; Hengge-Aronis, 1996) and the two-component signaling system *cpxA/R* (Bao et al., 2026), collectively constitute a putative complex regulatory network for the strain’s response to temperature stress.

The detection of IAA biosynthesis pathway genes indicates the strain’s potential to regulate plant growth. Analysis revealed a complete tryptophan biosynthesis operon (*trp*) (Chan et al., 1993), providing the precursor for IAA synthesis. Notably, key enzyme genes involved in multiple IAA synthesis pathways were identified, including *oxdA* (Yusfi et al., 2024) and *nthA/B* (Youseif et al., 2025) in the IAN pathway, *iaaM* (Gao et al., 2020) in the IAM pathway, and aldehyde dehydrogenase genes *aldA/B/H* (Shah et al., 2021; McClerklin et al., 2018; Guo et al., 2019) involved in the final steps of the IPA and TAM pathways. These findings suggest that YD4–4 may synthesize the plant hormone auxin through multiple metabolic routes.

The identification of genes related to nutrient conversion reveals the putative physiological basis of YD4-4’s plant growth-promoting effects from the perspective of nitrogen, phosphorus, and potassium nutrient conversion and utilization. In nitrogen metabolism, the genome encodes nitrate reductase complex genes (*narB*, *narG*, *narH*, *narI*, *narJ*) (Rubio et al., 1996; González et al., 2006; Blasco et al., 1992) and nitrite reductase genes (*nirB*, *nirD*) (Song et al., 2020; Yılmaz et al., 2022), indicating its potential ability to convert nitrate to ammonia, which may help improve plant nitrogen nutrition. Regarding phosphorus utilization, the strain possesses a high-affinity phosphate transport system (*pstS/C/A/B*) (Yuan et al., 2006), alkaline phosphatase genes (*phoR/B/U*) (Matilla et al., 2022), and an organic phosphorus degradation-related gene cluster (*phnA/N/W/X*) (Sosa et al., 2019; Phadtare et al., 1999), suggesting its capacity to mobilize soil phosphorus. The presence of potassium-related genes, such as the transport system *kdpA/B/C* (Huang et al., 2017), efflux system *kefA/B* (Büttner, 2008), and uptake systems *trkA/H* (Dosch et al., 1991; Kraegeloh et al., 2005) and *kup* (Tascón et al., 2020), provides a genetic explanation for its observed potassium-solubilizing activity on silicate media.

The presence of oxidative stress resistance and rhizosphere colonization-related genes imply its survival and functional performance in the plant rhizosphere environment. The genome contains a complete set of reactive oxygen species (ROS) scavenging systems, including catalases (*katA/B/E*, *srpA*) (Lee et al., 2005; Pan et al., 2017), peroxidases (*osmC*, *gpo*, *tpx*) (Lesniak et al., 2003; Dias et al., 2010; Comtois et al., 2003), superoxide dismutases (*sodA/B*) (Niederhoffer et al., 1990), and proline synthesis-related genes (*proA/B*) (Dandekar and Uratsu, 1988), which collectively mitigate oxidative damage. Additionally, genes associated with chemotaxis (*cheA/Y/W*) (Slightom and Buchan, 2009), flagellar assembly (*fliG/M/N*, *motA/B*) (Chevance and Hughes, 2008; Kubo et al., 2024), and the synthesis of organic acids (*actP*, *pycA*, *ldhA*) (Sheng et al., 2026; Yang D. et al., 2025; Zhou et al., 2003) and pyrroloquinoline quinone (*pqqB/C/D/E*) (Ge et al., 2015) are suggested to facilitate the strain’s directed migration toward plant roots and potential colonization capacity, as well as its ability to improve the soil microenvironment through metabolite secretion.

## 4 Discussion

### 4.1 Three PGPR strains improved *E. dahuricus* growth under low-temperature and drought stress

Cold and drought are key abiotic stresses constraining plant growth and distribution at high altitude (Sun et al., 2025; Magaña Ugarte et al., 2019). In this study, under simulated alpine conditions in low-temperature and drought pot experiments, *E. dahuricus* inoculated with PGPR strains demonstrated significant advantages in several aspects.

First, the notable biomass increase provides direct evidence for PGPR growth-promoting effects. These findings are consistent with extensive literature on the mechanisms of PGPR, which can directly stimulate cell division and elongation through the production of phytohormones such as IAA, and indirectly enhance plant growth by improving nutrient availability via processes such as phosphate solubilization and nitrogen fixation (Hernández-Amador et al., 2026; Mesele et al., 2026). The most effective growth promotion came from YD4-4. This result may be explained by two complementary factors. First, YD4–4 may directly regulate plant hormone levels by producing IAA and other phytohormones, promoting cell division and elongation. Second, through its phosphate-solubilizing activity, YD4–4 may convert unavailable soil phosphorus into available forms, providing adequate phosphorus and synergistically promoting growth. This supports Backer et al. (2018) that multifunctional synergy is key for efficient PGPR, whereas single-function strains often fail in complex soil environments.

Second, under low-temperature and drought stress, the beneficial effect of PGPR lies in maintaining the stability of the photosynthetic system (Zhang et al., 2023). The increase in chlorophyll a and b content following inoculation supports the role of PGPR in preserving photosynthetic capacity under adverse conditions. This agrees with Kim et al. (2015) that PGPR upregulate photosynthesis-related genes. It also aligns with Gowtham et al. (2022), where PGPR indirectly protect chloroplasts via antioxidant enhancement. Similarly, García-López et al. (2024) reported that PGPR-based biofertilizers reduced drought impact on strawberry net photosynthesis by approximately 67%, alleviating stomatal and biochemical limitations to protect photosynthesis. Our findings extend this understanding, confirming that such protection also applies to cold stress, suggesting that preserving photosynthetic machinery may be a universal PGPR strategy.

Third, under drought stress, the beneficial mechanisms of PGPR are manifested in the synergistic enhancement of osmotic adjustment and the antioxidant defense system (Sati et al., 2023). This study provides evidence that PGPR inoculation systematically improves plant drought resilience. The accumulation of proline directly alleviates water loss damage in protoplasts under dehydration stress such as drought (Avendaño et al., 2025). Concurrently, PGPR inoculation significantly activated protective enzymes (SOD, POD, CAT). This synergistic action scavenged excess ROS (Fujita and Hasanuzzaman, 2022; Chen et al., 2022), ultimately leading to reduced MDA (a membrane lipid peroxidation product) (Całyniuk et al., 2016). This result confirms that PGPR alleviates drought-induced oxidative membrane damage, aligning with recent findings that the antioxidant enzyme system plays an important role in combating drought-elicited oxidative stress (Turan et al., 2023).

However, the observed growth-promoting and stress-alleviating effects were obtained under controlled laboratory conditions using sterilized substrates, which do not fully replicate the complex biotic and abiotic interactions present in natural alpine meadows. Therefore, while our results demonstrate the clear potential of these PGPR strains under controlled environments, further investigation under field conditions on the Qinghai-Tibet Plateau is necessary to validate their practical efficacy and feasibility for alpine meadow restoration.

### 4.2 Comparative analysis of the isolated strains with previously reported PGPR strains

The genus *Pseudomonas* has been extensively documented as a PGPR with multiple beneficial traits, including phosphate solubilization, IAA production and so on (Sivasakthi et al., 2014). *Pseudomonas* sp. Bg32c, isolated from Moroccan phosphate mine, exhibited triple solubilization capabilities for phosphate, potassium, and zinc, in addition to synthesizing IAA (91 μg/mL) and displaying antagonistic activity against pathogens (Bouizgarne et al., 2023). *Pseudomonas* sp. PPR8k, isolated from the rhizosphere of *Phaseolus vulgaris*, demonstrated the ability to solubilize potassium, tricalcium phosphate (25 mg/mL), dicalcium phosphate (19 mg/mL), and zinc phosphate (21 mg/mL), while also producing IAA (39 μg/mL), ACC deaminase, and siderophores, and inhibiting the growth of multiple phytopathogenic fungi (Kumar et al., 2016). *Pseudomonas* sp. SM 8 and SM 20, isolated from the rhizosphere of *Minthostachys verticillata*, showed capacities to solubilize phosphates, produce IAA (557 ng/mL and 578 ng/mL, respectively), and exhibit high percentages of biocontrol through antibiosis (Meneguzzi et al., 2024). *Pseudomonas* sp. EU.26 and EU.9-4-2, isolated from alfalfa rhizosphere, possessed combined abilities of nitrogen fixation, phosphate solubilization, ACC deaminase activity, and siderophore production (Ünlü et al., 2024).

Regarding the genus *Lelliottia*, accumulating evidence has increasingly revealed its potential as a PGPR. *Lelliottia* sp. AS and BM have been shown to possess nitrogen fixation and IAA production capabilities (18.06 mg/mL and 20.35 mg/mL, respectively), with BM also demonstrating zinc solubilization activity (204.3 mg/mL) (Parashar et al., 2023). *Lelliottia* sp. JS-SCA-14 exhibited phosphate solubilization and siderophore production but did not show IAA production or ACC deaminase activity, while significantly increasing root and shoot length of tomato seedlings in pot experiments (Jeon et al., 2024). *Lelliottia* sp. MSR-M49, isolated from wheat rhizosphere, possessed combined traits of phosphate solubilization, IAA production (14.45 μg/mL), nitrogen fixation, and exopolysaccharide production, and promoted wheat growth in field trials (El-Akhdar et al., 2020).

The *Pseudomonas* sp. YD4-3, YD4-4, and *Lelliottia* sp. ND3–5 strains isolated in this study exhibited capacities for phosphate solubilization, potassium mobilization, and IAA synthesis, consistent with previously reported PGPR traits. However, direct numerical comparisons across different studies are not rigorous due to variations in experimental methodologies. Several functional traits reported in previous studies, including siderophore production, ACC deaminase activity, exopolysaccharide production, and pathogen antagonistic capacity, have not yet been investigated in our isolates. These unexplored characteristics provide clear directions for future in-depth investigations into the growth-promoting mechanisms and application potential of YD4-3, YD4-4, and ND3-5. In future work, we plan to systematically examine these traits to further elucidate the unique functional advantages of these plateau-derived strains.

### 4.3 Future perspectives based on the functional differentiation of PGPR strains

The three PGPR strains used in this study exhibited functional differentiation in promoting plant growth and inducing stress-resistant physiological responses. For instance, YD4–4 showed the most pronounced ability to enhance plant nitrogen uptake, which may be attributed to its unique nitrogen fixation or nitrogen mineralization capabilities. In contrast, YD4–3 achieved the greatest increase in root fresh weight in germination pouch assays. Meanwhile, ND3–5 significantly enhanced root activity and total phosphorus content under cold, and proline under drought. These distinct profiles reflect the functional diversity of PGPR communities. Such differentiation likely stems from variations in the unique metabolites secreted by each PGPR strain, such as phytohormones, ACC deaminase, and siderophores, or from the distinct signaling pathways activated during their interaction with host plants (Kesavardhini et al., 2025; Jalmi and Sinha, 2022). This functional divergence highlights the multifaceted application potential of PGPR resources from the Qinghai-Tibet Plateau and lays a solid foundation for subsequent investigations.

Building upon these findings, our future work will focus on translating these laboratory-based insights into practical applications for alpine meadow restoration. First, the rhizosphere colonization capabilities of the three strains will be systematically evaluated, including their survival rates in typical soil types of the Qinghai-Tibet Plateau, chemotactic responses to root exudates, and competitive colonization dynamics, so as to clarify the effective colonization windows and ecological adaptability of each strain in natural soil environments, thereby providing critical evidence for the subsequent optimization of composite inoculant formulations. Second, composite inoculant formulation will be developed by combining the three strains in various ratios, leveraging their complementary traits to achieve synergistic growth-promoting effects under complex field conditions (Ivanov, 2023). Third, field trials will be conducted across multiple degraded alpine meadow sites on the Qinghai-Tibet Plateau to evaluate the efficacy, persistence, and consistency of single and composite inoculations under natural environmental fluctuations. Fourth, metabolomic profiling of both bacterial cultures and inoculated plant tissues will be performed to identify the active metabolites, signaling molecules, and volatile organic compounds responsible for the observed physiological changes, thereby bridging the gap between genomic predictions and phenotypic outcomes. Fifth, the long-term ecological impact of biofertilizer application will be assessed, including their effects on soil microbial community structure, nutrient cycling, and ecosystem multifunctionality, to ensure both efficacy and environmental safety. Collectively, these future directions will systematically evaluate the feasibility of translating these plateau-derived PGPR strains into commercial biofertilizers, providing a comprehensive framework for the sustainable restoration of degraded alpine grasslands on the Qinghai-Tibet Plateau.

### 4.4 Possible IAA biosynthesis pathways in *Pseudomonas* sp. YD4-4

Whole-genome analysis of YD4–4 revealed numerous IAA biosynthesis-associated gene sequences. However, based on the currently accepted classification standards for IAA synthesis pathways, it is not yet possible to fully elucidate the IAA synthesis capacity of this strain. Taking the IPA pathway as an example, in this metabolic route, tryptophan is first catalyzed by an aminotransferase to form IPA, which is then converted to indole-3-acetaldehyde (IAAld) by indole-3-pyruvate decarboxylase. Finally, IAAld is converted to IAA by an aldehyde dehydrogenase (Tang et al., 2023). Although YD4–4 possesses the tan gene encoding an aminotransferase and the *ald* gene encoding a dehydrogenase, the key gene *ipdC* encoding indole-3-pyruvate decarboxylase in the IPA pathway was not annotated, rendering the IPA pathway incomplete. In the IAN pathway, IAN is converted to IAA by nitrilase, a key enzyme in this pathway (Tang et al., 2023). However, the *nit* gene encoding nitrilase (Normanly et al., 1997) was not annotated in the genome of YD4-4. Regarding the IAM pathway, although the key gene *iaaM*, encoding tryptophan monooxygenase which catalyzes the conversion of tryptophan to IAM, was annotated in the genome of YD4-4, the gene *iaaH* encoding indole-3-acetamide hydrolase was not annotated, and IAM requires catalysis by indole-3-acetamide hydrolase to be converted to IAA. In conclusion, based on the current classification criteria of IAA synthesis pathways, it is still impossible to fully clarify the IAA synthesis ability of the YD4–4 strain.

According to research by Duca et al. (2018) on *Pseudomonas* sp. UW4, they proposed a possible IAA synthesis pathway in strain UW4: a tryptophan precursor is first converted to indole-3-acetaldoxime (IAOx) by an unknown enzyme. Subsequently, phenylacetaldoxime dehydratase (Phe) converts IAOx to IAN. IAN is then converted to IAM by nitrile hydratase (NthAB), and finally, IAM is converted to IAA by an amidase (Ami). In this pathway, the genes encoding the related enzymes, *phe*, *nth*, and *ami*, were all annotated in the genome of strain YD4-4, suggesting that the IAA synthesis pathway in strain YD4–4 may be similar to that of strain UW4, which also belongs to the genus *Pseudomonas*.

It is speculated that this phenomenon may be related to the long-term adaptation of YD4–4 to the plateau environment. The Qinghai-Tibet Plateau and other extreme environments have characteristics such as low temperature, strong radiation, and low oxygen (Li et al., 2023). Microorganisms long-adapted to such environments often undergo genomic evolution, resulting in metabolic pathway differences from closely related mesothermal strains (Yan et al., 2016; Kumar et al., 2019). For example, 21 psychrophilic Cryobacterium strains from ice cores possess more stress response, motility, and chemotaxis genes than mesophilic family members, enabling extreme environment adaptation (Liu et al., 2020). Thus, the unconventional characteristics exhibited by YD4–4 in its IAA biosynthesis pathway may represent an adaptive strategy to the plateau environment. Under plateau cold conditions, the catalytic efficiency of conventional IAA pathway genes may be constrained (Ozimek et al., 2018), leading to their elimination or replacement by other enzymes under natural selective pressure. Consequently, YD4–4 may have gradually evolved alternative IAA biosynthesis pathways.

But it is important to emphasize that the functional assignments are derived from genome annotation, and they represent hypothetical inferences rather than experimentally confirmed mechanisms. While the presence of these genes provides a plausible genetic basis for IAA production in YD4-4, the actual expression, enzymatic activity, and regulatory control of these putative pathways remain to be experimentally validated.

### 4.5 Genomic evidence suggesting strain YD4–4 as a potential novel species

Comparative genomic analysis indicates that YD4–4 may represent a novel *Pseudomonas* species. Although the Average Nucleotide Identity (ANI) value based on FastANI algorithm between YD4–4 and its closest known relative, *Pseudomonas tritici*, was 95.267%, this falls within the recognized species cut-off of 95–96 % (Palmer et al., 2020), where definitive assignment is uncertain. More decisive evidence was provided by stringent genomic indices. The ANIb value calculated with the BLAST algorithm was 94.308%, below 95% ANI cutoff, which is the most frequently used standard for species demarcation (Jain et al., 2018). Crucially, the digital DNA-DNA hybridization (dDDH) value was 61.2%, which is substantially lower than the 70% boundary widely accepted for defining a bacterial species (Richter and Rosselló-Móra, 2009). The congruent results from these multiple, complementary genomic metrics consistently demonstrate significant divergence between YD4–4 and *P. tritici*. Therefore, despite being its closest phylogenetic neighbor in current databases, strain YD4–4 possesses a level of genomic distinctiveness that suggests it may be a novel species. However, the results of gene sequencing still require experimental verification. Before drawing a definitive conclusion, further comprehensive characterization, including phenotypic, biochemical, and phylogenomic analyses, is required to conclusively establish its taxonomic position.

## 5 Conclusions

Three growth promoting bacterial strains (*Pseudomonas* sp. YD4-3, *Pseudomonas* sp. YD4-4, and *Lelliottia* sp. ND3-5) were isolated from the *L. rotata* rhizosphere. Multidimensional experiments demonstrated their significant growth-promoting and stress-regulating effects on the important forage grass *E. dahuricus*.

Under low-temperature conditions, inoculation markedly enhanced biomass accumulation in *E. dahuricus*. Treated plants showed substantial improvements in aboveground and root growth over controls. Concurrently, chlorophyll synthesis and root metabolic activity were enhanced, and phosphorus uptake efficiency was significantly improved.

Under drought stress, the three strains enhanced host plant stress tolerance. They promoted the accumulation of the osmoregulatory substance proline, activated antioxidant enzyme systems including SOD, POD and CAT, and effectively mitigating drought-induced oxidative damage.

This study identifies microbial resources with both growth-promoting and stress-alleviating functions from a unique habitat, providing novel candidate strains and a genomic foundation for the application of microbial technologies in forage cultivation and ecological restoration in alpine regions.

## Data Availability

The datasets presented in this study can be found in online repositories. The names of the repository/repositories and accession number(s) can be found below: https://www.ncbi.nlm.nih.gov/, SAMN56667864.
