# Supplementary material for: Three plant growth-promoting rhizobacteria from the Qinghai–Tibet Plateau enhanced plant growth and alleviated low-temperature and drought stress
Source: Front Plant Sci. 2026 Sep 3;17:1907342. doi: 10.3389/fpls.2026.1907342 (PMC13582461; doi:10.3389/fpls.2026.1907342)
Supplement: Supplementary file 1 [file Table1.docx]

Supplementary Table 1 Potential genes responsible for plant growth promotion in YD4-4

| Categories | | Gene ID | Gene Name | Gene Annotation |
| --- | --- | --- | --- | --- |
| cold adaptation | cold shock protein | chr_1312 | *cspA* | cold shock protein |
|  |  | chr_4316 | *cspA* | cold shock protein |
|  |  | chr_1359 | *capB* | cold shock protein |
|  |  | chr_3782 | *cspD* | cold shock protein |
|  |  | chr_4066 | *capA* | cold shock protein |
|  |  | chr_4803 | *tapB* | cold shock protein |
|  |  | chr_4824 | *cspC* | cold shock protein |
|  | betaine | chr_818 | *opuBA* | osmoprotectant transport system ATP-binding protein |
|  |  | chr_819 | *opuCB* | osmoprotectant transport system permease protein |
|  |  | chr_820 | *opuCC* | osmoprotectant transport system substrate-binding protein |
|  |  | chr_5550 | *betB* | betaine-aldehyde dehydrogenase |
|  |  | chr_5551 | *betA* | choline dehydrogenase |
| synthesis of IAA | IAN pathway | chr_2930 | *nthB* | nitrile hydratase subunit beta |
|  |  | chr_2931 | *nthA* | nitrile hydratase subunit alpha |
|  |  | chr_2934 | *oxdA* | Haem-containing dehydratase |
|  | IAM pathway | chr_5464 | *iaaM* | amine oxidase |
|  |  | chr_5522 | *iaaM* | amine oxidase |
|  | acetaldehyde dehydrogenase | chr_2835 | *aldA* | lactaldehyde dehydrogenase / glycolaldehyde dehydrogenase |
|  |  | chr_2845 | *aldA* | lactaldehyde dehydrogenase / glycolaldehyde dehydrogenase |
|  |  | chr_2020 | *aldB* | aldehyde dehydrogenase |
|  |  | chr_5287 | *aldB* | aldehyde dehydrogenase |
|  |  | chr_2479 | *aldH* | 2,5-dioxopentanoate dehydrogenase |
| nitrogen fixation | nitrate reductase | chr_3356 | *narB* | assimilatory nitrate reductase catalytic subunit |
|  |  | chr_3589 | *narI* | nitrate reductase gamma subunit |
|  |  | chr_3590 | *narJ* | nitrate reductase molybdenum cofactor assembly chaperone NarJ/NarW |
|  |  | chr_3591 | *narH* | nitrate reductase / nitrite oxidoreductase, beta subunit |
|  |  | chr_3592 | *narG* | nitrate reductase / nitrite oxidoreductase, alpha subunit |
|  | nitrite reductase | chr_3354 | *nirD* | nitrite reductase (NADH) small subunit |
|  |  | chr_3355 | *nirB* | nitrite reductase (NADH) large subunit |
| phosphate solubilization | phosphate transport system | chr_3152 | *pstA* | phosphate transport system permease protein |
|  |  | chr_5911 | *pstA* | phosphate transport system permease protein |
|  |  | chr_3153 | *pstC* | phosphate transport system permease protein |
|  |  | chr_5912 | *pstC* | phosphate transport system permease protein |
|  |  | chr_3154 | *pstS* | phosphate transport system substrate-binding protein |
|  |  | chr_5913 | *pstS* | phosphate transport system substrate-binding protein |
|  |  | chr_5910 | *pstB* | phosphate transport system ATP-binding protein |
|  | phosphate transporter | chr_1415 | *pitA* | phosphate transporter |
|  |  | chr_4706 | *pitA* | phosphate transporter |
|  | alkaline phosphatase | chr_5909 | *phoU* | phosphate transport system protein |
|  |  | chr_5903 | *phoB* | two-component system, OmpR family, phosphate regulon response regulator PhoB |
|  |  | chr_5904 | *phoR* | two-component system, OmpR family, phosphate regulon sensor histidine kinase PhoR |
|  | Carbon-phosphorus hydrolase | chr_1627 | *phnX* | phosphonoacetaldehyde hydrolase |
|  |  | chr_3889 | *phnX* | phosphonoacetaldehyde hydrolase |
|  |  | chr_1799 | *phnN* | ribose 1,5-bisphosphokinase |
|  |  | chr_1913 | *phnR* | MFS transporter |
|  |  | chr_3888 | *phnW* | 2-aminoethylphosphonate-pyruvate transaminase |
|  |  | chr_5048 | *phnA* | protein PhnA |
| potassium mobilization | potassium transporter | chr_1707 | *kdpA* | potassium-transporting ATPase potassium-binding subunit |
|  |  | chr_1708 | *kdpB* | potassium-transporting ATPase ATP-binding subunit |
|  |  | chr_1709 | *kdpC* | potassium-transporting ATPase KdpC subunit |
|  | Potassium excretion system | chr_437 | *kefA* | potassium-dependent mechanosensitive channel |
|  |  | chr_1779 | *kefB* | monovalent cation:H+ antiporter-2, CPA2 family |
|  |  | chr_2553 | *kefB* | glutathione-regulated potassium-efflux system protein KefB |
|  | Potassium absorption system | chr_15 | *trkA* | trk system potassium uptake protein |
|  |  | chr_4770 | *trkH* | trk system potassium uptake protein |
|  |  | chr_1263 | *Kup* | KUP system potassium uptake protein |

Note: Categories: Processes related to promotion; Gene ID: Gene number; Gene Name: Recommended name from COG or Swiss-Prot annotation; Gene Annotation: Description of the annotated gene from KEGG or COG.
